# The Access, Initiation, Engagement, Retention, and Recovery (AIERR) Model: A Stage-Based Framework for Understanding Mental Health Service Utilization

**DOI:** 10.3390/healthcare14091212

**Published:** 2026-04-30

**Authors:** Cortney VanHook, Hyunjin Lee, Isaiah Ringo, Heather A. Jones

**Affiliations:** School of Social Work, University of Illinois Urbana-Champaign, Urbana, IL 61801, USA; hyl54@pitt.edu (H.L.);

**Keywords:** mental health service utilization, access to care, client engagement, treatment retention, recovery

## Abstract

**Background/Objectives:** Mental health service utilization gaps remain a persistent global public health challenge. Among the 61.5 million adults with any mental illness in the United States, nearly half went without treatment in the past year, and dropout rates from outpatient services among those who do enter care range from 19.7% to 30.8%. Only 30 to 60% of individuals with lifetime mental illness are in active recovery at any given time. Existing theoretical frameworks, including Andersen’s Behavioral Model, the Health Belief Model, and the COM-B framework, each address isolated phases of the care continuum but offer no unified structure for understanding the complete, sequential journey from first contact through sustained recovery. This article introduces the Access, Initiation, Engagement, Retention, and Recovery (AIERR) model to address this theoretical gap. **Methods:** A conceptual review was conducted following Hulland’s framework for theory development through narrative synthesis. Literature was identified through targeted searches in PubMed, PsycINFO, and Google Scholar, prioritizing peer-reviewed empirical studies, systematic reviews, and foundational theoretical frameworks. Sources were assigned to AIERR stages using predefined decision rules corresponding to each phase’s defining characteristics. **Results:** AIERR maps five sequential, interconnected stages: Access (structural, cultural, and systemic conditions enabling service reach), Initiation (the transition from provider identification to first appointment attendance), Engagement (active and meaningful treatment participation), Retention (sustained continuity of care), and Recovery (long-term reclamation of life quality and community belonging). For each stage, the framework identifies individual-level and structural-level barriers, facilitating conditions, and targeted intervention points. **Conclusions:** AIERR advances mental health services theory by unifying previously siloed frameworks, establishing stage-specificity as a core theoretical principle, and reorienting research and intervention strategy toward the upstream structural conditions that produce downstream utilization failures. These theoretical contributions require empirical testing to confirm. Implications for health equity research, clinical practice, and health systems design are discussed.

## 1. Introduction

Despite decades of investment in treatment development, the majority of individuals who need mental health care never receive it, and a substantial proportion of those who do begin treatment do not stay long enough to benefit. The problem is not primarily a failure of treatment development, effective interventions exist across virtually every diagnostic category, but a failure of delivery. According to the most recent National Survey on Drug Use and Health among the 61.5 million adults with any mental illness, nearly half, 47.9%, went without treatment [1]. Treatment receipt remains low across racial groups, and income compounds the problem further: cost and inadequate insurance coverage consistently rank among the most commonly reported barriers to care, with uninsured and low-income adults facing the steepest obstacles to accessing services [2,3]. Among those who do enter treatment, the problem does not end at the door. Dropout rates from outpatient services range from 19.7% to 30.8% [4]. The causes are both individual and structural, including ineffective treatment matching, negative provider interactions, and systemic barriers that accumulate across the care process [5,6]. Addressing this failure requires more than better treatments or increased funding. It requires a theoretical framework capable of mapping the full utilization continuum, identifying where and why breakdown occurs at each phase, and generating the stage-specific intervention logic that generic approaches have consistently failed to provide.

Despite growing recognition of these challenges, existing frameworks for understanding mental health service utilization often address isolated components rather than the full continuum of care. For instance, Andersen’s [7] Behavioral Model provides robust insights into predisposing factors, enabling resources, and need that drive access, yet it offers limited guidance on sustaining participation once services begin. Similarly, the Health Belief Model [8] explains initial help-seeking decisions but does not extend to the processes of engagement, retention, or long-term recovery. Pescosolido’s Network Episode Model [9] illuminates the social embeddedness of service initiation, while the Capability, Opportunity, Motivation, and Behavior (COM-B) framework [10] helps explain behavior change within treatment, but neither comprehensively addresses the sequential stages clients navigate from first contact through sustained recovery. The Connectedness, Hope, Identity, Meaning, and Empowerment (CHIME) framework [11] and Substance Abuse and Mental Health Services Administration [12] Recovery Model provide valuable perspectives on recovery-oriented care, yet they primarily focus on the final stages rather than the earlier access and initiation hurdles many clients never overcome. While each of these models contributes essential understanding, no single framework currently captures the complete, sequential journey from gaining access to achieving sustained recovery. This gap leaves educators without a unified structure for teaching the full spectrum of service utilization, and it leaves practitioners without a comprehensive roadmap for identifying where interventions are most needed along the care continuum.

**Figure 1:**
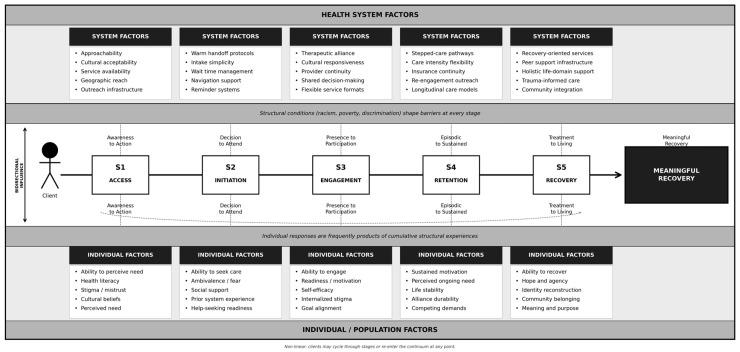
The AIERR Model: A Stage-Based Framework for Understanding Mental Health Service Utilization Adapted from the Levesque et al. [13] Patient-Centered Access Framework. Note. The figure depicts a client (**left**) progressing through five sequential stage gates toward meaningful recovery (**right**). At each gate, health system factors (**upper panel**) interact with individual and population factors (**lower panel**) to determine whether stage transition occurs. Bolded labels between gates identify the key transition each stage represents. Dashed vertical connectors indicate bidirectional influence between system and individual levels. Shaded horizontal banners denote that structural conditions, including racism, poverty, and discrimination, shape barriers at every stage and that individual-level responses are frequently downstream products of cumulative structural experiences. The curved arc (lower pathway) indicates that stage progression is non-linear; clients may cycle through or re-enter the continuum at any point. AIERR = Access, Initiation, Engagement, Retention, and Recovery.

To address this gap, this article introduces the Access, Initiation, Engagement, Retention, and Recovery (AIERR) model: a stage-based theory-generating framework, developed by the authors through synthesis of implementation science, health services research, and clinical literature, that maps five distinct yet interconnected stages of mental health service utilization (Figure 1). The AIERR model is a newly proposed framework that has not yet undergone formal empirical validation; this article represents its first full scholarly presentation. By integrating and extending existing single-stage frameworks, AIERR proposes a unified structure for identifying where utilization breaks down, why it breaks down at each stage, and what conditions may support successful progression through the continuum.

The five stages of the AIERR model progress sequentially through the care continuum while acknowledging that clients may cycle through stages non-linearly. Stage 1, Access, addresses whether individuals can realistically reach services given structural, informational, cultural, and systemic barriers. Stage 2, Initiation, captures the transition from identifying a provider to attending a first appointment. Stage 3, Engagement, examines active, meaningful participation in treatment. Stage 4, Retention, examines sustained continuity of care over time. Stage 5, Recovery, addresses the long-term journey toward reclaiming quality of life, identity, and community connection beyond symptom reduction. Each stage carries distinct barriers, facilitators, and intervention targets. This review will (1) present the AIERR model and define each stage, (2) synthesize the literature on barriers and facilitators at every phase, (3) demonstrate how AIERR integrates and extends existing frameworks, and (4) identify implications for research, practice, and health systems design.

## 2. Methodology

This conceptual review follows Hulland’s [14] framework for theory development through literature synthesis. Hulland distinguishes conceptual reviews from systematic reviews: rather than cataloguing and statistically synthesizing empirical findings, conceptual reviews use the existing literature as raw material for constructing or extending theoretical understanding, requiring transparency about synthesizing logic and the future testability of the resulting framework. Consistent with this approach, we reviewed extant knowledge on mental health service utilization, identified tensions and gaps across existing models, and synthesized these insights into the AIERR framework, a reconceptualization that integrates previously disparate theoretical perspectives into a unified, stage-based model.

### 2.1. Literature Selection

Relevant literature was identified through targeted searches in PubMed, PsycINFO, and Google Scholar, with additional sources drawn from the reference lists of key papers and recent reviews. Core search terms included “mental health service utilization,” “help-seeking behavior,” “treatment initiation,” “treatment engagement,” “treatment dropout,” “treatment retention,” “mental health recovery,” and “barriers to mental health care,” applied individually and in combination. Inclusion criteria prioritized: peer-reviewed articles, systematic reviews, conceptual frameworks, and policy documents published in English; relevance to one or more phases of the service utilization continuum; and theoretical or empirical contribution to understanding barriers, facilitators, or stage-specific intervention targets. Sources were excluded when their primary focus was clinical symptom outcomes rather than the utilization process itself, or when they addressed only pediatric or specialty populations outside the adult mental health context.

The selection emphasized foundational theoretical models (e.g., the Behavioral Model of Health Service Use, the Health Belief Model, the Network Episode Model), landmark reviews, and contemporary research on barriers and facilitators across the utilization continuum. Substantially more sources were reviewed than ultimately cited; sources not included were either superseded by more methodologically rigorous work addressing the same construct, or were insufficiently central to the framework’s development to warrant citation.

Consistent with Hulland’s [14] framework, transparency in this conceptual review is achieved through the empirical grounding of the literature selected to establish the framework, rather than through the reproducible search procedures that characterize systematic reviews. Given the conceptual nature of the paper, the goal is not for exhaustive coverage but identifying theoretical relevance and influential contributions to inform framework development.

### 2.2. Conceptual Synthesis

We employed a narrative synthesis process. Literature was assigned to AIERR conceptual domains using the following decision rules: (1) studies addressing service availability, geographic reach, financial access, or help-seeking initiation prior to first contact were coded to Access; (2) studies addressing first appointment attendance, intake completion, or the transition from service identification to actual participation were coded to Initiation; (3) studies addressing therapeutic alliance, active treatment participation, and session-to-session involvement were coded to Engagement; (4) studies addressing dropout prevention, continuity of care, and long-term treatment maintenance were coded to Retention; and (5) studies addressing post-treatment outcomes, wellness maintenance, and life domain recovery beyond symptom reduction were coded to Recovery. Where studies spanned multiple stages, they were analyzed under the stage most central to their primary research question. Within each domain, the synthesis prioritizes identification of barriers, facilitating conditions, and intervention targets. We also mapped relationships between concepts and identified recurring themes relevant across models and populations to identify mechanisms and recurring themes across stages.

## 3. Model Characteristics

The AIERR model conceptualizes mental health service utilization through five distinct yet interconnected stages (Figure 1), each representing both a unique phase of the service experience and a potential point for targeted intervention or system improvement. From a health systems perspective, this stage-based structure provides clear targets for quality improvement initiatives, resource allocation decisions, and systematic evaluation of service delivery effectiveness.

The AIERR model is distinguished by three theoretical features. First, it is client-centered: success at each stage is defined by client goals and experiences, not solely by provider or system metrics, making the model applicable across diverse populations and settings. Second, it is explicitly bidirectional between individual and structural levels: barriers at every stage operate simultaneously at the level of the individual (psychological readiness, perceived need, cultural beliefs) and the structural level (system design, insurance architecture, provider availability), and the model requires analysis of both. Third, it is intervention-specific: for each stage, AIERR identifies distinct leverage points where targeted action is most likely to support progression through the continuum, enabling researchers to design stage-matched studies and practitioners to select strategies suited to where a client is struggling. This combination positions the AIERR model as a potentially valuable tool for research and practice. The model is intentionally transdiagnostic. The utilization barriers and stage-specific mechanisms AIERR identifies, including structural access failures, initiation ambivalence, engagement erosion, and retention disruption, operate across DSM-5 diagnostic categories, subthreshold presentations, and broader psychosocial distress rather than being specific to any single disorder. While the expression of these mechanisms will vary by diagnosis, chronicity, and severity, such that a person navigating first-episode depression faces a meaningfully different utilization journey than someone managing a serious and persistent mental illness, the framework advances the position that the underlying stage structure and the categories of barriers and facilitators it identifies provide a valid organizing foundation across these variations. Disorder-specific applications and adaptations are an expected and necessary direction for future work.

Each AIERR stage is defined by a distinct operational criterion that marks when that stage begins and ends. Access is complete when the structural, informational, and cultural conditions necessary for realistic service reach are sufficient for a given individual. Initiation is complete when the client attends their first appointment and begins the intake process. Engagement is present when the client is actively and meaningfully participating in treatment sessions, not merely attending. Retention is present when the client maintains treatment continuity across the care episode, sustaining connection long enough for clinically meaningful change to occur. Recovery is an ongoing, nonlinear process of reclaiming life quality, identity, and community belonging, with or without complete symptom resolution.

The model acknowledges that many barriers, including stigma, cultural mistrust, weak therapeutic alliance, and financial strain, operate across multiple stages rather than within one. This is not a conceptual weakness; it reflects an empirical reality that AIERR is explicitly designed to capture. The framework does not claim stage-exclusive mechanisms. Rather, it claims that the primary causal pathway through which a given mechanism produces utilization failure differs by stage. Stigma that prevents help-seeking operates primarily as an Access barrier; stigma that causes a client to conceal information from their provider operates primarily as an Engagement barrier; stigma that corrodes hope and self-concept operates primarily as a Recovery barrier. The same construct produces different failure modes at different points in the continuum, and stage-specific analysis is what makes those distinctions visible. The practical implication is that an intervention targeting the wrong stage is unlikely to work, not because the intervention is ineffective in principle, but because it is addressing a mechanism that is not the primary driver of failure at that point in the continuum. Stage-matched intervention, not generic quality improvement, is therefore what the framework recommends and what empirical testing should evaluate.

### 3.1. Stage 1: Access to Services

Access represents the foundational stage where opportunity meets feasibility, which is the point at which individuals can realistically obtain care that meets their needs. Access operates across multiple dimensions simultaneously. Penchansky and Thomas [15] conceptualize access through six critical domains: availability (sufficient services exist), accessibility (geographic/physical reach), accommodation (service organization fits client life), affordability (cost within means), acceptability (cultural/personal fit), and awareness (knowledge of options). A service may score well on some dimensions while failing critically on others, and a single failure point can block access entirely.

**Figure 2:**
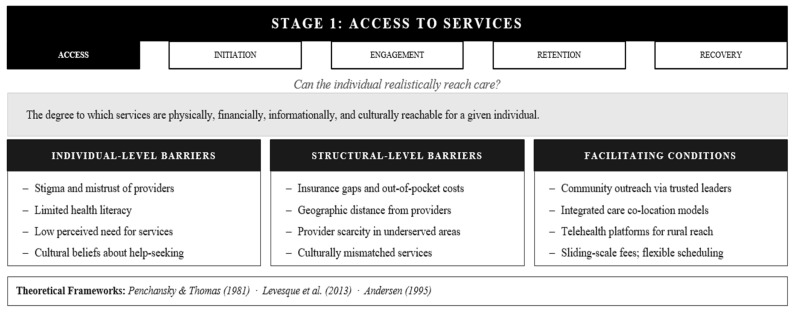
The AIERR Model, Stage 1: Access to Services—Barriers, Facilitators, and Integrated Theoretical Frameworks. Note. Individual-level barriers are frequently dowstream products of structural conditions. AlERR = Access, Initiation, Engagement, Retention, and [7,13,15].

Levesque et al. [13] deepen this understanding by highlighting the dynamic interaction between health system characteristics and individual abilities. Systems must not only provide approachable, acceptable, available services, but individuals must possess the ability to perceive need, seek help, reach services, pay for care, and engage once there. This bidirectional model reveals why access failures cannot be reduced to either system deficits or individual limitations; rather, both must align. Andersen’s [7] Behavioral Model adds the critical recognition that access depends on the interplay of predisposing factors (demographics, beliefs), enabling resources (insurance, social support, community resources), and need (perceived and evaluated). Taken together, the frameworks reviewed here make clear that access is not a binary state but a complex, multidimensional phenomenon that requires simultaneous alignment across structural, informational, cultural, and individual conditions (Figure 2).

The barriers that constrain access operate at both individual and structural levels, clustering into four reinforcing domains. At the structural level, transportation difficulties, geographic distance, financial constraints, insurance gaps, and limited service availability prevent physical and economic reach [16]. These barriers are concrete rather than abstract: a potential client may find that no in-network provider practices within a reasonable distance, that clinic hours end before the workday does, or that the nearest facility is technically accessible on a map but functionally unreachable without a car. Such obstacles are not indicators of disinterest in care; they are accurate assessments of a system that has not removed the practical conditions that make seeking help possible.

Informational barriers include low health literacy, language differences, and limited awareness of available resources. Such barriers operate at both individual and system levels and leave people unable to navigate what exists even when services are theoretically nearby [17,18]. When a potential client is unaware that a community mental health center exists two miles from their home, that reflects a failure of outreach and system design rather than individual disengagement. Cultural and psychological barriers, including stigma, mistrust, and negative past experiences, deter help-seeking at the individual level, yet these are themselves shaped by structural experiences of racism and historical mistreatment of marginalized communities [19]. For clients from communities with sustained histories of institutional harm, concerns about whether a provider will understand their experience or treat them with dignity are not irrational. Such concerns reflect accumulated individual and community-level experiences with systems that have historically failed or harmed people who look like them [20,21]. Finally, systemic design barriers such as rigid appointment scheduling, multi-step intake processes, and culturally mismatched services signal to potential clients that this system was not designed with their needs in mind [22]. When a caller is placed on a long hold, presented with a complex intake questionnaire, or encounters a provider unfamiliar with their community, that signal is powerfully reinforced. Addressing access requires simultaneous attention to all four domains; resolving one while leaving others intact rarely produces meaningful improvement.

What works to improve access? Community outreach that deploys trusted local leaders builds both awareness and acceptability, particularly in communities where historical mistreatment has generated legitimate mistrust [23]. Integrated care models that embed mental health services in primary care or schools address accommodation and accessibility by meeting people where they already are [24]. Digital health platforms can dramatically improve accessibility for rural populations while offering flexible accommodation for scheduling constraints [25], though they introduce new barriers for those without reliable internet or digital literacy. Culturally and linguistically tailored services do not just improve acceptability; they signal a fundamental repositioning of who belongs in mental health care, often simultaneously addressing awareness through community networks [26]. Transportation assistance, sliding-scale fees, walk-in and flexible scheduling, and streamlined intake processes remove concrete obstacles while demonstrating organizational commitment to true accessibility [27,28]. These evidence-based strategies demonstrate that access barriers, while formidable, are not insurmountable when systems commit to implementing multifaceted solutions that address the diverse needs of the communities they serve.

The access stage reveals a fundamental insight for health systems and researchers: good intentions are insufficient. A system can genuinely want to serve all who need it while remaining systematically inaccessible through the compounding effects of structural design, cultural misalignment, and resource constraints. The individual who appears disengaged from the help-seeking process has often encountered not one access barrier but multiple operating simultaneously, each individually surmountable but collectively overwhelming. Addressing access therefore requires sustained, multi-domain commitment rather than isolated fixes, as resolving a single barrier while leaving others intact rarely produces meaningful improvement.

### 3.2. Stage 2: Initiation of Mental Health Services

**Figure 3:**
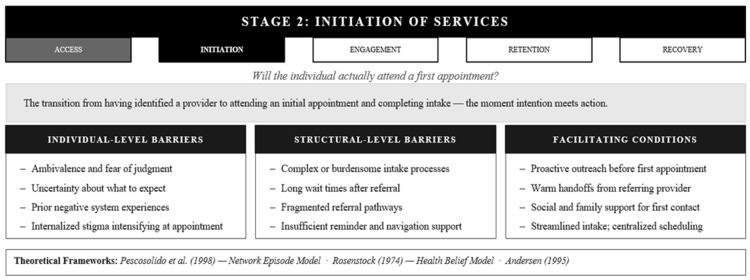
The AIERR Model, Stage 2: Initiation of Services—Barriers, Facilitators, and Integrated Theoretical Frameworks. Note, Access and initiation are theoretically distinct. Full access is necessary but not sufficient—initiation is governed by a sparateset of psychological social, and structural mechanisms. AIERR = Access, Initiation, Engagement, Retention, and Recovery [7,8,9].

Access and initiation are not synonymous, though they are often conflated in both research and practice. Having a scheduled first appointment does not ensure a client will attend; knowing how to reach a clinic does not mean someone will walk through the door. Initiation represents the critical transition from having access to attending services, which captures the moment when theoretical accessibility becomes actual participation [29]. This stage captures the first concrete steps: attending an initial appointment, completing an intake, and beginning treatment. Here, intention meets action, and the gap between them explains much of the attrition in mental health service utilization (Figure 3).

Three theoretical frameworks illuminate why this transition proves so difficult for many. Pescosolido’s Network Episode Model reveals initiation as fundamentally social, as it is embedded in relationships with family, friends, and community members who may encourage, discourage, or actively prevent someone from starting care [9]. A mother whose own family dismisses mental health treatment as weakness faces social headwinds even after identifying a provider. The Health Belief Model [8] explains that initiation requires individuals to perceive both susceptibility and severity (“I have a problem that matters”), recognize benefits (“treatment will help”), believe barriers can be overcome (“I can actually do this”), and encounter a cue to action that catalyzes movement. Ambivalence at any of these points stalls initiation. Andersen’s [7] Behavioral Model adds that even with access achieved, initiation depends on enabling resources, particularly practical supports like childcare, transportation, and time off work, and on how need is perceived and evaluated by both the individual and their social network. These frameworks converge on a crucial insight: initiation is where psychological readiness, social context, and practical capability must align.

The barriers that derail initiation often catch even well-designed access efforts off guard. Someone may complete a screening, receive a referral, and schedule an appointment, only to never arrive. Psychological barriers including fear of judgment, uncertainty about what to expect, stigma, and ambivalence intensify as the appointment approaches [30,31]. Previous negative experiences with health systems create anticipatory dread, as prior encounters characterized by dismissiveness or cultural misalignment become powerful deterrents to trying again [32,33]. Social barriers emerge when family discourages attendance, cultural beliefs frame mental health care as inappropriate, or concerns about confidentiality within small communities create genuine risk [32]. Practical barriers that seemed surmountable during scheduling, such as transportation, childcare, or work conflicts, become insurmountable the morning of the appointment [34,35]. Process barriers like complicated intake paperwork, long wait times, confusing referral pathways, or inadequate communication about what to expect create friction at the exact moment when motivation is most fragile [36,37]. These combined barriers explain why so many individuals who successfully navigate access never complete the critical transition to actual service initiation.

What moves people across the initiation threshold? Proactive outreach, warm welcomes, and clear information about what to expect help transform a daunting step into a manageable one [38]. Social support from family, friends, or peers who encourage attendance can be decisive, particularly in cultural contexts where individual decisions are family decisions [31]. Streamlined intake, flexible scheduling, reminder systems, and concrete help with transportation or childcare reduce friction [39,40]. System-level approaches, including integrated care models, warm handoffs where the referring provider personally introduces the client, and centralized intake, all support successful initiation [41]. Together, these supports communicate that the step is not only possible but actively facilitated by the system designed to serve the client.

The initiation stage clarifies a critical theoretical distinction: access and utilization are not equivalent. A person can have complete access to a service, including insurance, geographic proximity, and cultural acceptability, and still never attend a first appointment. This gap between access and initiation reflects the operation of a distinct set of psychological, social, and practical mechanisms that existing access-focused frameworks do not fully capture. Referral is not enough; the transition from identified need to first contact requires deliberate structural and relational support that the access stage alone cannot provide.

### 3.3. Stage 3: Service Engagement

**Figure 4:**
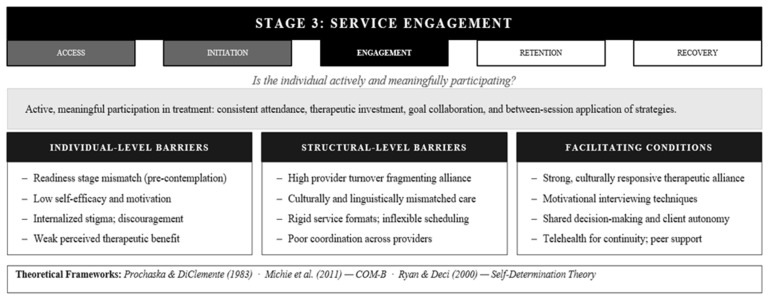
The AIERR Model, Stage 3: Service Engagement—Barriers, Facilitators, and Integrated Theoretical Frameworks. Note. Low engagement signals misalignment between system design and client needs, not client failure COM-B = Capability, Opportunity, Motivation, and Behavior. ATERR = Access, Initiation, Engagement, Retention, and Recovery [10,43,45].

Attendance is a readily measurable proxy, but as a standalone metric it is theoretically insufficient: it captures physical presence without addressing whether the conditions necessary for clinical change, including active investment, therapeutic collaboration, and meaningful participation, are occurring. Engagement transcends physical presence; it represents active, meaningful participation: showing up consistently, investing in therapeutic activities, collaborating on goals, applying strategies between sessions, allowing vulnerability, and doing the difficult work that change requires [42]. This stage captures the difference between showing up and showing up fully. It is the stage where treatment actually happens, making it perhaps the most critical phase for clinical outcomes, yet also the most fragile, as engagement fluctuates constantly based on internal states, external circumstances, and relationship quality (Figure 4).

Understanding engagement requires recognizing its dynamic, multifaceted nature. The Stages of Change model [43] positions engagement as movement through readiness phases. Someone may be physically present but still in precontemplation or contemplation, not yet ready for the action stage engagement requires. The COM-B framework [10] reveals engagement as the product of three intersecting forces: capability (having the skills and knowledge to participate), opportunity (external circumstances enable participation), and motivation (want and need align to drive participation). When any one element fails, engagement falters. The Patient Health Engagement Model [44] captures how clients’ sense of their own empowerment and agency evolves through engagement; it is not static but a developing capacity that providers must actively cultivate. Self-Determination Theory [45] adds the critical insight that sustained engagement depends on meeting three fundamental psychological needs: autonomy (feeling choice, not coercion), competence (experiencing effectiveness and growth), and relatedness (feeling genuinely connected to the provider). Across all these frameworks, the therapeutic relationship emerges as the engine of engagement, as strong, trusting, culturally responsive connection is what makes active participation possible and sustainable.

Engagement erodes through multiple pathways. Logistical obstacles, including transportation breakdowns, schedule conflicts, and caregiving crises, disrupt participation; when such disruptions recur, they erode the momentum that sustained engagement requires [46,47]. Organizational factors such as high provider turnover, poor service coordination, and inflexible systems further undermine sustained participation [48,49]. Psychological and relational barriers are often most potent: ambivalence, low motivation, internalized stigma, and weak therapeutic alliance, specifically when clients do not feel truly heard or understood, remove the relational foundation that sustains difficult work [50,51,52]. Clients who comply with attendance requirements while feeling unseen or misunderstood are physically present but relationally absent, a form of surface-level compliance that does not produce the outcomes associated with genuine engagement. Cultural mismatches, linguistic barriers, and perceived microaggressions create guardedness rather than the openness engagement requires [53]. The cumulative effect explains why many clients drift away after a few sessions having attended but never fully engaged.

What sustains engagement through its inevitable fluctuations? The quality of the therapeutic relationship stands paramount. Providers who communicate with genuine empathy, validate client experiences without judgment, maintain cultural humility rather than claiming cultural competence, and consistently demonstrate that they see the client as a person rather than a diagnosis or case, build the relational foundation that sustains engagement through difficulty [54,55]. Clients need to feel that requests for a different approach or honest feedback about what is not working will be met with flexibility rather than rigidity. This relationship does not emerge automatically from professional credentials; it requires ongoing intentional cultivation. Beyond the dyadic relationship, social supports from family, peers, and community organizations reinforce participation and normalize the engagement process [56]. System-level facilitators include flexible scheduling that adapts to life’s unpredictability, telehealth options that maintain connection when transportation fails, user-friendly communication methods, and services genuinely tailored to cultural and linguistic needs [57,58]. Treatment approaches that honor client autonomy through shared decision-making, that adapt methods to individual and cultural preferences rather than imposing standardized protocols, and that explicitly recognize and celebrate progress, even incremental gains, communicate respect and build investment [59,60]. Digital tools for between-session connection and consistent check-ins when engagement seems to waver demonstrate organizational commitment to sustaining participation [61,62]. Across all these facilitators, the common thread is that engagement is not something clients sustain alone; it is something systems and providers must actively maintain.

The engagement stage represents a fundamental reorientation in how utilization is theorized. Traditional utilization models focused on access, specifically whether people entered services, but engagement reveals that entry is necessary but not sufficient. What matters is whether clients are actively and meaningfully participating once inside the system. The AIERR framework reframes low engagement not as client failure but as evidence of misalignment between system design, provider behavior, and client needs, recognizing that sustained participation emerges from the dynamic interaction between what clients bring to treatment and what systems and providers make possible.

### 3.4. Stage 4: Retention of Services in Mental Health Care

**Figure 5:**
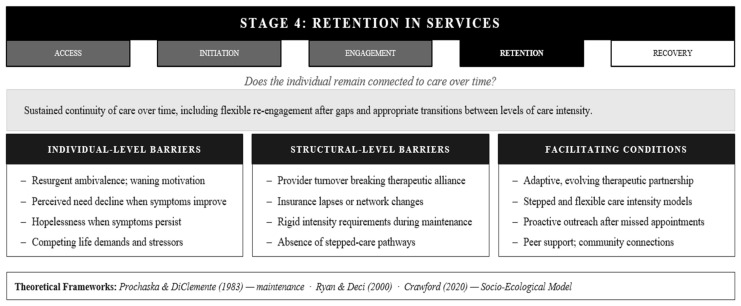
The AlERR Model, Stage 4: Retention in Services Barriers, Facilitators, and Integrated Theoretical Frameworks. Note. Retention is theoretically distinct from engagement, driven by longitudinal sustainability and provider continuity rather than participation quality alone. AIERR Access, Initiation, Engagement, Retention, and Recovery [43,45,66].

Engagement and retention, while related, capture different dimensions of service utilization: engagement concerns the quality and depth of participation within treatment contact, while retention concerns its duration and continuity over time. Where engagement measures how actively and meaningfully clients invest, retention measures whether that contact is sustained [63,64]. A client can be highly engaged during sessions yet drop out after two months; conversely, a client can be retained in services for years with varying levels of engagement. Consider a client who enters weekly therapy, engages meaningfully, and improves sufficiently to transition to biweekly and then monthly sessions, attending each one consistently—the intensity of treatment has changed substantially, but retention is intact. Retention, as the framework conceptualizes it, is not synonymous with indefinite or constant-dose treatment; clients are within their right to terminate, and brief or single-session interventions are efficacious for some populations [65]. Rather, retention refers to sustaining connection long enough for clinically meaningful change to occur, flexibly returning after gaps without penalty, and transitioning to other appropriate supports when intensive services are no longer needed, all while maintaining the thread of connection to care. Understanding retention requires recognizing that sustained connection is not the same as sustained intensity, and that clients navigate ongoing internal questions about whether continued participation remains viable as their circumstances and needs evolve (Figure 5).

Theoretical frameworks reveal retention as fundamentally about sustainability through change. The Stages of Change model addresses maintenance and relapse patterns [43]; retention is what allows people to consolidate gains and navigate setbacks without complete disengagement. Andersen’s [7] Behavioral Model highlights how enabling resources and perceived needs shift over time; what enabled retention initially may no longer suffice as life circumstances evolve. Self-Determination Theory reminds us that autonomy, competence, and relatedness needs do not get met once and remain satisfied; rather, they require ongoing nurturance through changing contexts [45]. The Socio-Ecological Model positions retention within nested, dynamic systems, including individual, relational, organizational, community, and policy contexts, all of which fluctuate and continuously reshape the forces supporting or undermining sustained connection [66]. This multi-level, temporal view reveals retention as an active process of adaptation rather than passive inertia.

Why do clients who were successfully engaged nevertheless leave prematurely? Practical obstacles that were manageable initially, such as transportation, childcare, and financial strain, compound over time, particularly if circumstances worsen [67,68]. Insurance coverage lapses or provider network changes can abruptly sever established therapeutic relationships, leaving clients without a clear pathway back to care even when they intend to return [49,69]. Perceived need shifts: symptom improvement frequently triggers premature termination, while persistent symptoms generate hopelessness about continuing, both representing distinct failure modes that require different clinical responses [70,71]. Psychosocial factors, including resurgent ambivalence, provider relationship ruptures, and self-criticism around setbacks, further corrode commitment [72,73]. For marginalized clients, accumulated microaggressions and cultural disconnection progressively drain the will to return [74,75]. Approximately one-fifth of adults drop out before completing the recommended treatment course [76]. Understanding why retention fails, and at which mechanism, is prerequisite to designing systems capable of sustaining connection across the full arc of recovery.

What sustains connection through months and years? Fundamentally different strategies than those supporting engagement prove necessary. The therapeutic relationship remains foundational but must evolve; what began as rapport-building must deepen into genuine partnership capable of weathering disagreements, setbacks, and changed circumstances [77,78]. Providers who signal flexibility by adapting the treatment approach as the client’s life circumstances change rather than insisting on a fixed protocol communicate the kind of responsiveness that sustains long-term connection. Provider continuity becomes increasingly important; repeated transitions damage retention by forcing clients to rebuild trust repeatedly. Flexible, responsive care, including stepped care models and assertive community treatment that adapts frequency and format to changing needs (more intensive during crisis, less intensive during stability, with easy pathways to increase again), communicates that retention does not mean rigid adherence to a fixed protocol [79,80]. Providers should routinely assess practical barriers as a standard component of ongoing clinical contact, recognizing that circumstances change and that a client who managed transportation and scheduling demands initially may be quietly struggling with those same demands three months later without raising them unprompted [81]. Proactive outreach after missed appointments, robust follow-up systems, and use of technology for between-session connection prevent gaps from becoming permanent departures [62]. The social relationships surrounding a client between sessions, including peers, family, and community networks, can motivate retention in ways that clinical contact alone cannot; a trusted person in a client’s life who affirms the value of staying in care often carries more persuasive weight than the provider delivering it [82]. Critically, systems must recognize and celebrate progress, not just symptom reduction but life improvements, relationship strengthening, and capacity building, making retention feel worthwhile even when dramatic change is not immediately apparent [59]. These retention-supporting strategies collectively communicate that the treatment relationship can grow and adapt alongside the client’s evolving life circumstances, transforming what might otherwise be a rigid, time-limited intervention into a flexible resource clients can draw upon across the arc of recovery.

Retention asks a question that utilization research has historically underspecified: not whether someone enters treatment, but whether the conditions exist for them to remain connected over time. The question is most consequential for individuals living with serious and persistent mental illness, for whom care is not a time-limited intervention but an ongoing relationship with a system that must remain navigable across years of fluctuating symptoms, functional demands, and life circumstances. Retention requires that the system remains accessible and responsive as a client’s needs evolve, that gaps do not become permanent exits, and that stepping down in treatment frequency is experienced by the client not as being pushed out of care but as a meaningful marker of progress within it. A client who takes a reprieve of several weeks and returns to the same organization, therapist, or treatment regimen has not failed at retention. Neither has the client who transitions from weekly therapy to monthly check-ins, or who moves from formal treatment to peer support while maintaining connection to the broader care system. In both cases, a meaningful threshold of retention has been reached, sufficient to sustain the continuity of care that treatment progress requires.

### 3.5. Stage 5: Recovery

**Figure 6:**
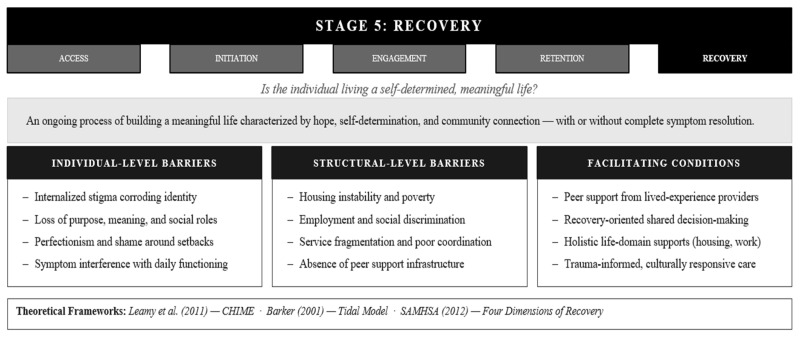
The AIERR Model, Stage 5: Recovery—Barriers, Facilitators, and Integrated Theoretical Framework. Note. Recovery reframes the terminal outcome of service utilization: not symptom elimination, but a self-determined life of meaning, agency, and community connection CHIME = Connectedness, Hope, Identity, Meaning, Empowerment. AIERR = Access, Initiation, Engagement, Retention, and Recovery [11,12,85].

Recovery represents the most misunderstood stage of the AIERR framework, not because the concept is obscure, but because it has been simultaneously medicalized and romanticized in ways that obscure its actual nature. Recovery is not synonymous with cure, symptom freedom, or a fixed destination reached and then permanently occupied. Rather, recovery is an ongoing process of building a meaningful life characterized by hope, self-determination, and community participation, with or without complete symptom resolution [83,84]. Importantly, recovery does not begin where retention ends. A client can be actively retained in treatment while simultaneously making recovery-level gains in identity, hope, and community belonging. What marks the transition from retention as the primary clinical concern to recovery as the organizing framework is not discharge but the emergence of sustainable functioning, a point at which the client’s capacity to build and maintain a meaningful life no longer depends primarily on active treatment contact, even if ongoing connection to care continues in a reduced or supportive form. Consider a client with a history of serious depression who has been in weekly therapy for eighteen months, has returned to meaningful employment, rebuilt key relationships, and developed a reliable set of coping strategies, yet continues monthly check-ins with their provider. That client is simultaneously retained in care and in recovery. The monthly contact is no longer the primary vehicle of change; it is the scaffolding that supports a life that has already been substantially reclaimed. This definition, drawn from decades of recovery movement advocacy and research, fundamentally reorients how successful mental health service utilization is conceptualized. The orienting question shifts from clinical symptom status to whether individuals are living a life they find worthwhile, a reframing with direct implications for how outcomes are measured, how services are designed, and how system success is evaluated (Figure 6).

Contemporary recovery frameworks illuminate this transformation-focused understanding. The CHIME framework identifies five interconnected elements central to personal recovery: connectedness to others and community; hope about the future and possibility of change; identity beyond illness, reclaiming valued roles; meaning and purpose in life; and empowerment through personal control and responsibility [11]. Notably, symptom severity appears nowhere in this framework, not because symptoms do not matter, but because recovery is defined by life quality, not clinical metrics [11]. The Tidal Model centers the client’s narrative and lived experience, insisting that recovery must be defined by the person living it, not by professionals observing it [85]. The Recovery Star provides practical tools for collaboratively tracking progress across life domains, including relationships, living skills, work, identity, and well-being, making visible the multidimensional nature of recovery [86]. The Substance Abuse and Mental Health Services Administration [12] model organizes recovery around four dimensions: health (managing symptoms), home (safe stable place to live), purpose (meaningful activities), and community (relationships and social connection), all underpinned by person-driven, trauma-informed principles. Together, these frameworks make clear that recovery is fundamentally about reclaiming life, not just managing illness.

What constrains recovery? The barriers operate at fundamentally different levels than those impeding earlier stages. Structural inequities, including poverty, homelessness, unemployment, and discrimination, create conditions where merely surviving dominates, leaving little space for the forward-looking work recovery requires [87]. Social isolation and lack of meaningful relationships undermine the connectedness recovery demands [88]. Pervasive stigma, both external (others’ judgments) and internalized (self-judgment), corrodes hope and identity, making it difficult to envision oneself as more than one’s diagnosis [89]. When internalized stigma narrows a person’s sense of possible futures, foreclosing aspirations around employment, relationships, or community belonging, it operates as a direct structural barrier to the identity reclamation that recovery requires. Internal psychological barriers including perfectionism that frames setbacks as catastrophic failures, shame that prevents reaching out, and fear of the vulnerability necessary for growth further impede recovery’s developmental process [90]. A client in recovery may require only maintenance level contact, yet that minimal connection often represents more than it appears. It is the stable framework within which hard-won progress has been sustained, and its disruption can have consequences disproportionate to its apparent intensity [91]. Changes in personal circumstances, such as relocation or loss of insurance coverage, and changes within the system itself, such as the dissolution of a peer support group, the departure of a trusted provider, or organizational restructuring, can leave a client in recovery without the relational and structural anchors that have supported their progress [48]. At this stage, such disruptions carry particular weight: the client is not in crisis, but the scaffolding that has quietly sustained their functioning is gone, and finding one’s footing without it can be genuinely disorienting, even for someone who has made substantial gains. These challenges, whether structural, psychological, or systemic, amplify for marginalized populations facing compounded discrimination and reduced access to recovery-supporting resources, revealing how recovery barriers extend far beyond the clinical realm to encompass fundamental questions of identity, belonging, and the right to envision and pursue a meaningful life.

What facilitates recovery? Hope, understood as the belief that change is possible and one’s past does not determine one’s future, is foundational [92]. Peer support from others with lived experience provides this hope through living proof that recovery is real [93,94]. Supportive relationships with family, friends, and community combat isolation and reinforce positive identity [95]. Recovery-oriented services that practice shared decision-making, honor client expertise, and support self-advocacy position clients as drivers of their own recovery [91]. Holistic supports, including stable housing, meaningful work, and financial stability, recognize that recovery happens in daily life, not only in therapy sessions [96]. Systems that honor each person’s self-defined vision of a meaningful life create space for authentic transformation rather than mere symptom management [12]. Across all of these facilitators, the common thread is that recovery is not something that happens to a client inside a clinical setting; it is something a client builds in the context of relationships, community, and daily life, with systems playing a supporting rather than a directing role.

The recovery stage asks a fundamental theoretical question: what does successful utilization actually mean? If the answer is symptom reduction and discharge, then the preceding four stages are sufficient. But recovery-oriented frameworks, including CHIME, SAMHSA’s Recovery Model, and the Tidal Model, converge on a different answer: successful utilization means the client is living a self-determined life with hope, meaning, and community connection, with or without complete symptom resolution. Incorporating recovery as the terminal stage of AIERR reframes the entire framework: the purpose of access, initiation, engagement, and retention is not treatment compliance but the creation of conditions for recovery as the client defines it. A framework that ends at retention asks whether the client stayed. A framework that ends at recovery asks whether the client flourished.

## 4. Discussion

The AIERR model makes three theoretical contributions to the mental health service utilization literature. First, it provides integration across previously siloed frameworks. Existing models each illuminate a portion of the care continuum: Andersen’s Behavioral Model explains access, the Health Belief Model explains initial help-seeking, Pescosolido’s Network Episode Model illuminates initiation, COM-B explains engagement dynamics, and CHIME and SAMHSA’s Recovery Model describe sustained recovery. Each is stage-specific, and single-model approaches have consistently produced inconsistent findings because different mechanisms operate at different points in the continuum [97]. AIERR does not replace these frameworks; it provides the integrative structure that connects them, allowing researchers and practitioners to trace how mechanisms at one stage shape the vulnerabilities that define the next. This integration extends to recent developments in digital mental health, where expanding modalities of access and between-session connection have introduced new stage-specific dynamics that existing frameworks are not structured to capture [25,61]. Second, AIERR introduces stage-specificity as a theoretical principle, generating the testable proposition that stage-mismatched interventions, such as applying access-focused strategies to a retention problem, may account for a portion of intervention inefficacy in the existing literature. Third, AIERR repositions the individual-structural relationship across the continuum, mapping how structural conditions produce individual-level barriers and recognizing that responses such as ambivalence, low perceived need, and premature dropout are frequently downstream products of structural inequalities rather than independent personal characteristics [98]. Together, these three contributions position AIERR not as a replacement for existing utilization theory but as the integrative scaffold that existing theory has lacked, one that connects established frameworks, specifies where and why breakdown occurs at each phase, and generates the stage-specific logic that research and practice have not yet had a unified structure to support.

### 4.1. Implications for Research and Practice

For researchers, the AIERR model generates a structured set of empirical questions. Each stage transition represents a testable prediction: that the predictors of successful initiation differ from those of successful retention, and that stage-targeted interventions are expected to outperform generic service improvement strategies. The model also generates predictions about sequential dependencies, including whether access barriers increase initiation vulnerability and whether engagement quality moderates the relationship between retention duration and recovery outcomes. These propositions are testable through longitudinal administrative data, sequential mixed-methods designs, pragmatic trials, and interrupted time series analyses of stage-targeted interventions. Empirical testing will require observable indicators capable of capturing stage-specific status and transition. At Access, candidate indicators include provider-to-population ratios within a defined geographic area, insurance acceptance rates, wait time to first available appointment, and scores on validated structural barrier screening instruments such as the Barriers to Access to Care Evaluation [99]. At Initiation, relevant indicators include time elapsed between referral receipt and first appointment attendance, no-show rates for scheduled intake appointments, warm handoff completion rates, and dropout occurring between scheduling and first attendance [41]. At Engagement, candidate indicators include session attendance consistency, between-session task completion rates, and therapeutic alliance scores such as the Working Alliance Inventory [100]. At Retention, appropriate indicators include treatment episode duration, provider continuity indices measuring the number of provider transitions within a care episode, time elapsed between a missed appointment and next system contact, and the proportion of treatment episode endings that are planned rather than unplanned. The Barriers to Retention Scale offers a candidate measure for retention-specific barriers, though adaptation for mental health populations is warranted given its origins in substance use treatment contexts [101]. Successful step-down from higher to lower intensity services represents an additional positive retention indicator, consistent with the framework’s conceptualization of sustained connection as distinct from sustained intensity. At Recovery, candidate indicators include community participation indices, functional domain scores across housing, employment, and relationships, and CHIME-based self-report measures capturing connectedness, hope, identity, meaning, and empowerment [11]. These indicators are proposed as starting points for operationalization rather than a definitive measurement battery, and selection will appropriately vary by population, setting, and research question.

For practitioners and health systems, the model proposes a framework for precision assessment and intervention planning. Rather than responding to service failures with generic quality improvement efforts, practitioners may use the stage structure to assess where in the continuum a client is struggling and select interventions with demonstrated efficacy at that stage. The model reconceptualizes dropout not as client failure but as potential evidence of system-level misalignment with client needs, a reframing consistent with social work’s person-in-environment perspective [102]. For health systems, the stage structure may support quality monitoring that extends beyond aggregate utilization rates to examine where specific populations are disproportionately losing contact with care. These are theoretical and design-based contributions; systematic empirical testing is required before applied implementation claims can be made.

### 4.2. Limitations

Several limitations must be acknowledged. First, as a conceptual literature review, this work synthesizes broad themes rather than providing systematic methodological rigor or quantitative evidence synthesis. The framework rests on narrative integration of diverse evidence rather than meta-analytic precision. Second, the AIERR model was developed with sustained outpatient mental health treatment as its primary referent; its applicability to short-term interventions, medication management, and crisis services may require stage-level adaptation and represents a direction for future theoretical development. Third, while AIERR employs stage-based language, the framework is more precisely understood as a continuum model in which the five phases represent analytically distinct but empirically overlapping domains; future theoretical work should address the extent to which strict stage-model assumptions, including discreteness and invariant sequencing, hold across populations and settings. Fourth, the literature reviewed is predominantly Western, limiting immediate applicability to diverse global contexts where health system infrastructure, cultural factors, and community resources differ markedly. Fifth, the focus on adult mental health services limits direct applicability to pediatric and geriatric populations, where service utilization processes differ due to developmental factors and family involvement patterns. Sixth, stage definitions derived from the same literature used to populate their content carry inherent circularity risk, and independent operationalization and measurement development represents a necessary next step before empirical testing. Seventh, while the model comprehensively maps service utilization barriers, the degree to which individual practitioners can address them varies considerably. Structural obstacles such as insurance policy, transportation infrastructure, and poverty require advocacy and collective action beyond individual clinical practice, and educators using the AIERR model should help students distinguish between barriers addressable through direct practice and those requiring macro-level intervention.

## 5. Conclusions

The AIERR model advances mental health services theory by providing what has been missing from the utilization literature: a unified framework capable of mapping the complete journey from first contact through sustained recovery. The persistent gap between mental health need and service use is not a problem that any single stage-specific model can resolve, because the mechanisms that prevent access differ from those that derail initiation, which differ again from those that erode engagement, fragment retention, and constrain recovery. By integrating previously siloed frameworks into a sequential, stage-specific structure, AIERR generates a coherent set of testable propositions, a common conceptual vocabulary for cross-study synthesis, and a practical logic for matching interventions to the point in the continuum where they are most needed. The framework is offered not as a finished product but as a theoretical foundation, one that invites empirical challenge, culturally adaptive modification, and the kind of sustained scholarly engagement that transforms a promising heuristic into an evidence-based tool for improving population mental health.

## Data Availability

No new data were created or analyzed in this study.
